# A prospective multicenter European study on flexible ureterorenoscopy for the management of renal stone

**DOI:** 10.1590/S1677-5538.IBJU.2015.0528

**Published:** 2016

**Authors:** Francesco Berardinelli, Silvia Proietti, Luca Cindolo, Fabrizio Pellegrini, Roberto Peschechera, Hennessey Derek, Orietta Dalpiaz, Luigi Schips, Guido Giusti

**Affiliations:** 1 Dipartimento di Urologia, “S. Pio da Pietrelcina ‘’ Hospital, Vasto (CH), Italia;; 2 Centro di pietra presso il Dipartimento di Urologia, Humanitas centro clinico e di ricerca, Rozzano, Italia;; 3 Department of Urology, Craigavon Area Hospital, Portadown (UK);; 4 Urologische Klinik, Medizinische Universität Graz, Austria

**Keywords:** Ureteroscopy, Kidney Calculi, Lithotripsy, Laser

## Abstract

**Purpose:**

The aim of this study was to describe the outcomes and the complications of retrograde intrarenal surgery (RIRS) for renal stones in a multi-institutional working group.

**Materials and Methods:**

From 2012 to 2014, we conducted a prospective study including all RIRS performed for kidney stones in 4 European centers. Demographic information, disease characteristics, and perioperative and postoperative data were gathered. Patients and stone data, procedure characteristics, results and safety outcomes were analyzed and compared by descriptive statistics. Complications were reported using the standardized Clavien system.

**Results:**

Three hundred and fifty-six patients underwent 377 RIRS with holmium laser lithotripsy for renal stones. The RIRS was completed in all patients with a mean operative time of 63.5 min. The stone-free status was confirmed endoscopically and through fluoroscopic imaging after the first procedure in 73.6%. The second procedure was performed in twenty patients (5.6%) achieving an overall stone free rate of 78.9%. The overall complication rate was 15.1%. Intra-operative and post-operative complications were seen in 24 (6.7%) and 30 (8.4%) cases, respectively.

**Conclusions:**

RIRS is a minimally invasive procedure with good results in terms of stone-free and complications rate.

## INTRODUCTION

The management of kidney stones has evolved radically over the years. Previously, extracorporeal shockwave lithotripsy (SWL) and percutaneous nephrolithotomy (PCNL) were the preferred treatment modalities for renal calculi. The impressive technologic improvement in endoscopic flexible equipment with the recent advent of digital technology made ureteroscopic approach to kidney calculi evolve from a mere diagnostic tool to a real operative procedure capable to treat the vast majority of renal stone.

Several studies have demonstrated the advantages and high success rates of PCNL in the management of larger renal stones. However, experiences with RIRS have revealed comparable stone free rate (SFR) with less risk of renal damage and bleeding (1). Recently, it has been demonstrated that retrograde intrarenal surgery (RIRS) can be a good management option for mid-sized stones between 2 to 4cm (2, 3) and in special circumstances such as pregnancy, anatomic malformations or coagulopathy and solitary kidney (4, 5) although it still not is the standard of care.

In 2013, the European Association of Urology’s (EAU) Guidelines on Urolithiasis for the first time listed RIRS as a viable treatment option for all kidney stones, including stones larger than 2cm in diameter, in experienced hands in high-volume centers (6). It is noteworthy, that for stones smaller than 2cm, SWL is no longer considered mandatory as first approach so that indications to RIRS have massively been broadened.

However, RIRS is associated with some disadvantages being the possible need for staged procedures one of the major. Secondly, risk of ureteral injuries and the costs of acquisition and maintenance of the complex endourological armamentarium are other concerns that might have been limited the capillary diffusion of this endoscopic procedure (7). Herein we describe a multi-center prospectively study of RIRS for renal stones whose aim is to highlight the clinical outcomes with particular attention to the complications rate.

## MATERIALS AND METHODS

### Clinical Data

A prospective study of all patients who underwent RIRS for kidney stone disease in four European referral centers was performed from 2012 to 2014. Data were recorded prospectively on each patient. Patient data obtained included: age, sex, body mass index (BMI), history and physical examination findings, specific comorbidities, American Society of Anesthesiologists (ASA) class risk. The stone parameters evaluated were: the number of stones, stone location, previous treatments for stone, stone diameters and stone composition. Double-J stent preoperatively, congenital renal anomalies (pelvi-ureteric junction obstruction, horseshoe kidney), anticoagulant therapy were evaluated.

Stone location was classified as renal pelvis/ureteropyelic junction, superior/middle/inferior major calyces and multiple caliceal location. The preoperative assessment included non-contrast computed tomography (CT) or KUB (kidney-ureter-bladder) plain radiograph and renal ultrasound (US).

An antibiotic therapy, either as prophylaxis with cephalosporin or fluoroquinolone or adapted 5 days antibiotic therapy in patients with an intraoperative positive urine culture was administered. Informed consent was obtained from all patients, and the possible need for a staged procedure in order to obtain satisfactory stone clearance was mentioned. Exclusion criteria were as follows: pregnancy and cachexia.

### Operative and Postoperative Data

The operative time was defined as the time that passed from insertion into the urethra of the cystoscope/semirigid ureteroscope for introducing the guidewire to the completion of ureteral stent placement. All the RIRS were performed using different types of flexible ureteroscopes: Flex-X2 or Flex-XC (Karl Storz Endoscope, Germany), the URF-P5 or URF-V (Olympus Europe, Germany). Lithotripsy was achieved by means of 200/273μm Holmium laser fibers. A guidewire was placed in the upper urinary tract through a rigid cystoscope or semirigid ureteroscope under fluoroscopic guidance. According to surgeon preference, visual assessment of the ureter and ureteropelvic junction was performed with the semirigid ureteroscope. Alongside a second safety guidewire an Ureteral Access Sheath (UAS) (Flexor 9.5/11.5Fr or 12/14Fr, Cook Medical Bloomington, IL, USA, Navigator 11/13Fr, Boston Scientific, Natik, MA, USA or Retrace 10/12Fr, Coloplast/Porges Humlebæk, Denmark) up to the proximal ureter was placed. If the UAS placement was impossible, a sheathless procedure was attempted. If this last attempt failed, a pigtail, double J ureteral catheter (DJ) was left in situ for passive dilatation and the procedure was delayed.

A tipless nitinol basket was used to extract the fragments and to re-position lower pole calculi to a more accessible calyx prior to intracorporeal lithotripsy. The procedure was concluded after stone-free status was confirmed by both ureteroscopic inspection and fluoroscopy (leaving only ungraspable gravel or fragments <2mm) or in case of bleeding or at the decision of the surgeon. By the end of the procedure, the ureter was ureteroscopically and fluoroscopically assessed for possible lesions. The ureteral injuries were classified in major and minor (8). The classification of ureteral wall injuries proposed by Traxer et al. (9) was not used as it was not available at the time of the beginning of the series. A DJ was applied at the end of procedure according to surgeon preference or after complicated procedures.

### Follow up

The ‘‘stone-free’’ status was defined as no evidence of stones or stones less than 2mm on one-month postoperative CT and/or KUB and/or US, prescribed following the surgeon preferences. Patients with residual fragments, requiring a further RIRS, were routinely scheduled for the second treatment 30-45 days following the previous procedure and were evaluated at 4 weeks from the last procedure. All patients were followed up to 6 months, with serial plain radiograph or renal ultrasound. Postoperative complications were assessed according to the modified Clavien classification (10).

## RESULTS

### Clinical Data and Patient Characteristics

Three hundred and fifty-six patients underwent 377 RIRS with holmium lasertripsy for renal stones. The cohort included 226 (63.5%) male and the mean age was 53.5 years (SD: 14.3). One third of patients (34.3%) had a history of urolithiasis, and the 32% already received a previous treatment. In 64% of patients we found a single stone (mean diameters: 12.4x9.5mm) and in 165 cases (46%) the stone was located in the pelvis/ureteropyelic junction. Demographic data and clinical characteristics of the cohort are listed in Tables 1 and 2.

**Table 1:** Demographic data and clinical characteristics.

	N or mean	% or ±SD
**Gender**		
Male	226	63.5%
Female	130	36.5%
Age, years	53,5	±14.3
BMI, kg/m^2^	26.5	±4.2
ASA score	1.6	±0.8
Diabetes	41	11.5%
CKD	10	2.8%
Hyperuricemia	13	3.7%
Other metabolic disease	26	7.3%
Renal malformation	30	8.4%
Previous stone treatments	114	32.0%
Hydronephrosis	152	42.7%

**Values are mean and SD or absolute number and percentage.BMI** = Body Mass Index; **CKD** = chronic Kidney disease; **ASA** = American Society of Anesthesiologists class risk.

**Table 2:** Operative and postoperative data.

	N or mean	% or ±SD
**Operative time (min)**		
UAS	63.5	±32.4
Number of stone	283	79.5%
- Single	228	64.1%
- Multiple	128	35.9%
**LOS**	2	±2
**Stone size**		
- length (mm)	12.5	±5.3
- width (mm)	9.4	±4.5
**Stone location**		
- upper calyx	9	2.5%
- mild calyx	29	8.1%
- lower calyx	58	16.2%
- pelvis	92	25.8%
- UPJ	73	20.5%
- pelvis + calyx	79	22.1%
- multiple calyces	34	9.5%
**Stone composition**		
- Ca oxalate	164	46%
- Uric acid	16	4.5%
- Mixed (Ca oxalate+Uric acid)	28	7.8%
- Brushite	43	12%
- Struvite	7	2%
- Cystine	18	5%
- Other	7	2%
- Unknown	92	25.8%
Pre-operative stent or nephrostomy	77	21.6%
**Stone X-ray characteristics**		
- radiopaque	274	76.9%
- radiolucent	90	23.1%
Post-operative stenting	332	93%
Re-intervention	20	5.5%
**SFR**		
- First procedure	262	73.6%
- Second procedure	281	78.9%

Values are mean and SD or absolute number and percentage. **N** = number; **LOS** = length of stay; **UPJ** = ureteropyelic junction; **SFR** = stone free rate; **Ca** = calcium.

### Operative and Postoperative Data

RIRS was safely completed in all patients with a mean operative time of 63.5 min (range 13–250 min). The mean number of procedures per patient was 1.05. The UAS was placed in 283 patients (79.5%), among these the 65% received a large UAS (11/13FR or 12/14F or larger) and the remaining 35% a smaller one (9.5/11.5F or 10/12F).

In 262 cases (73.6%) a complete stone-free status was confirmed endoscopically and through fluoroscopic imaging. Postoperatively a DJ was positioned in 332 patients (93.2%). Twenty patients (5.6%) with a greater diameter than the overall average (mean diameters: 13x9.9mm) received a second retreatment and 1 of these (0.2%) required a third treatment. The second procedure achieved complete stone clearance for 281 patients, for an overall SFR of 78.9%. Complete operative and postoperative data are provided in Table-2. If we analyze the subgroup of lower pole stone and sheatless procedures, the SFR is 68.9% and 77.4%, respectively.

### Complications

The overall complication rate was 15.1%. Intra-operative and post-operative complications were experienced in 24 (6.7%) and 30 (8.4%) cases, respectively. A detailed description of the complications and the action taken are showed in Table-3. No major ureteral injuries occurred. Minor ureteral wall injuries were noted in 11 patients (3%) and managed successfully with a stent placement (for 3 to 6 weeks) (mucosal abrasion not reported). Two patients (0.5%) were re-admitted following discharge from hospital with non obstructive pyelonephritis: they were treated with intravenous antibiotics and bladder catheter. Two patients (0.5%), left unstented after the procedure, required DJ insertion after readmission for an obstructive pyelonephritis. No complications higher than Clavien grade IIIa were observed. No patients complained late complications to follow-up visit after 6 months.

**Table 3:** Overall Complications according to Clavien classification.

	Intraoperative		Postoperative	

Complication	Patients, N (%)	Action	Patients, n (%)	Action
**Clavien Grade I**				
Hematuria	7 (1.9)	Fluid irrigation	2 (0.5)	DJ placement
Lumbalgia	-	-	1 (0.2)	Analgesics
Fever	-	-	23 (6.4)	Antipyretics
**Clavien Grade II**				
Perforation of pelvis/ calyx	5 (1.4)	DJ placement	-	-
Ureteral injury	11 (3)	DJ placement	-	-
Non obstructive pyelonephritis	-	-	2 (0.5)	Antibiotics
**Clavien Grade IIIa**				
Obstructive pyelonephritis	-	-	2 (0.5)	DJ placement Antibiotics

**Total**	**24 (6.7)**	**30 (8.4)**		

**N** = number; **DJ** = double J stent; **Clavien grade I** = Any deviation from the normal postoperative course without the need for pharmacological treatment or surgical, endoscopic and radiological interventions. **Clavien grade II** = Requiring pharmacological treatment with drugs other than such allowed for grade I complications. **Clavien III**= complicaiton requiring surgical, endoscopic or radiological intervention.

## DISCUSSION

This prospective multi-institutional study on RIRS for renal calculi has shown this approach to have excellent stone clearance rates with an acceptable complication profile. Major technical and surgical developments in endoscopic technologies and technique for the treatment of urolithiasis have led to changes in treatment approach, and subsequently to international guidelines (6). In this large study, we have demonstrated that the SFR achieved after the first treatment of RIRS to be as high as 73.6%. This finding is comparable to similar studies that demonstrated SFR of 65-79% (11-15).

We also noted that only 20 patients (5.6%), with a mean diameter greater than the overall average, required a second look RIRS to ensure the stone-free status. We do not regard this as a treatment failure of the first procedure, but rather as a necessary part of a planned staged procedure to ensure stone clearance, in particular for large renal stones (eg. stones>2cm). This strategy allowed us to achieve an overall SFR equal of 79%.

Over the last 10 years, RIRS has become an increasingly important option for the treatment of the majority of kidney stones even in the most complicated clinical scenarios such as pregnancy, obesity, coagulopathy, large renal stones, calyceal diverticula, and kidney malformations (4). RIRS is well accepted by patients, the convalescence is minimal, it generally does not require a prolonged hospital admission (2 days) or prolonged absence from work (2). In addition, using the endoscopic approach to the kidney, reduces the risk of blood loss, renal parenchyma damage and renal impairment. In contrast, in recent years we have seen a drift away from SWL and PCNL. This is in part due to SWL providing a low SFR (16) and a high re-treatment rate for large stones and for stones located in the lower pole calyx (17). Similarly, PCNL that is the gold standard for large kidney stones and is more effective than SWL for lower pole stones (18) is characterized by a risk of serious complications (19, 20). We noted that 77 patients (21.6%) had DJ stent placement prior to RIRS. DJ placement may facilitate passage of the UAS and extraction of the fragments (21), however this does not justify the routine DJ insertion before surgery except in case of a septic obstructed upper urinary tract or in case of ureter stricture that will not make possible the passage of access sheath (22). In contrast to this, a post-operative DJ stent was left in situ in 332 patients (93%). This is done to facilitate drainage, prevent post-operative ureteral obstruction. In 79% of cases the DJ stents were placed for 30 days or less. The uses of the UAS can significant facilitate RIRS and stone clearance by allowing multiple entry and reentry to the kidney, decreasing intrarenal pressure and protect the scope from damage (23, 24). However, the routine use of a UAS is matter of debate (6). In this study, an UAS was used in 80% of the cases. The size of the UAS varied according to clinical conditions. In 52% of the cases a larger UAS was employed. This probably reflects the surgeon’s preference for lasertripsy. If the surgeon has a predilection for fragmentation and stone extraction, the preferred setting of laser has been low frequency/high energy using medium/large diameter sheath. This allows the extraction of slightly larger fragments. If the surgeon prefers to dust by vaporization the preferred setting of laser is high frequency/low energy using a medium/small UAS sufficient. However, the choice between vaporization and fragmentation is not only related to the surgeon preferences but also by stone size. For bigger stone could be better to start with the vaporization of the outer part of stone moving to the fragmentation of the residual part and the extraction of fragments.

The overall 30-day complication rate in this study was 15.1% being 6.7% intraoperative and 8.4% post-operative. These data are according with the data of EAU guidelines on Urolithiasis that report an overall complication rate of 9-25% (6). It’s noteworthy that the complications rate of pure RIRS is lacking. In fact, the complication rate reported by EAU guidelines are based on surgical series of semirigid ureteroscopy for ureteral stone. A recent meta-analysis that included 2 randomized and 8 non-randomized studies showed an overall complication rate of RIRS of 10.4%. It was concluded by the authors, that the interpretation of complications proved to be challenging because of a lot of key information (blood transfusion, antibiotic use, definition for sepsis, need for preoperative stenting, definition of ureteral injury, timing of post-operative stenting, etc.) were not clearly stated (25). The complication rate in our study, that at first analysis could be considered slightly higher than expected, should be considered as a viable finding. In fact, in this prospective and standardized setting, we considered any deviation from normal post-operative course as a complication and described it according to Dindo-modified Clavien classification (26).

Some limitations for this study should be acknowledged. The first limitation of this multicenter study is the different imaging modalities used to assess SFR. While CT is the most sensitive modality for assessing residual fragments, logistical and cost reasons may prohibit its routine use (27, 28). Ultrasonography was used frequently as a postoperative imaging test based on its low cost, ready accessibility, and lack of radiation. Lack of uniform evaluation of residual fragment might also represent a weakness. However, even if a central residual fragment evaluation ideally increases validity by minimizing the interobserver variability, it is useless, from a clinical viewpoint, since variability is common in clinical practice (4). A second limitation would be the non-uniform treatment approach and the different experience of the surgeons. The multi-institutional nature of our cohort may be interpreted as limitation, however we believe that, in order to evaluate the generalizability of these findings, a certain grade of heterogeneity in baseline characteristics, rather than homogeneity, is advisable and desirable.

In our opinion, it is important to remind that, as stated by Giusti et al. (4), the “key to success is avoiding the start of RIRS on your own. Furthermore, detailed and frank counselling of the patients is strongly encouraged to inform them not only about the minimal invasiveness but also about outcomes of the surgeons/centers and the potential for staged multiple procedures in the most difficult cases and the possibility, although rare, of major complications”.

## CONCLUSIONS

This European multicenter prospective study confirmed that RIRS performed with the newest generation of technical equipment allowed us to achieve a very high SFR without compromising on safety. Nevertheless, cost of acquisition and maintenance and reimbursement policies by national health systems represent a critical issue that should be resolved to allow RIRS become a routine procedure available in all urological departments and not just in a few tertiary centers.

## ARTICLE INFO

Int Braz J Urol. 2016; 42: -86
